# Characterization of *Mycobacterium tuberculosis* strains in Beijing, China: drug susceptibility phenotypes and Beijing genotype family transmission

**DOI:** 10.1186/s12879-018-3578-7

**Published:** 2018-12-14

**Authors:** Yi Liu, Xuxia Zhang, Yuqing Zhang, Yong Sun, Cong Yao, Wei Wang, Chuanyou Li

**Affiliations:** Department of Bacteriology and Immunology, Beijing Key Laboratory on Drug-Resistant Tuberculosis Research, Beijing Tuberculosis and Thoracic Tumor Research Institute/Beijing Chest Hospital, Capital Medical University, Beijing, 101149 China

**Keywords:** *Mycobacterium tuberculosis*, Beijing genotype, Prevalence, Drug resistance, Association

## Abstract

**Background:**

The most prevalent strains of *Mycobacterium tuberculosis* (*M.tb*) in Beijing belong to the Beijing genotype family. The influence of Beijing genotype prevalence on the development of drug resistance, and the association of infection with Beijing genotype *M.tb* with population characteristics, in Beijing, however, are still unclear.

**Methods:**

In this retrospective study, 1189 isolates were subjected to drug susceptibility testing (DST) and molecular epidemiological analysis, and differences in the percentage of drug resistance between Beijing and non-Beijing genotype strains were compared. The association between the occurrence of drug resistance and the prevalence of Beijing genotype *M.tb* was analyzed using statistical methods.

**Results:**

The Beijing genotype family was the dominant genotype (83.3%) among the 1189 *M.tb* isolates. Beijing genotype *M.tb* strains were more likely to spread among males [*p* = 0.018, OR (95% CI):1.127(1.004–1.264)] and people in the 45–64 age group [*p* = 0.016, OR (95% CI): 1.438 (1.027–2.015)]. On the contrary, non-Beijing genotype *M.tb* strains were more probably disseminated among the over 65 [*p* = 0.005, OR (95% CI):0.653 (0.474–0.9)] and non-resident population [*p* = 0.035, OR (95% CI):1.185(0.985–1.427)]. DST results showed that 849 (71.4%) strains were fully sensitive to first-line drugs, while 340 (28.6%) strains were resistant to at least one drug, and 9% (107/1189) were MDR-TB. The frequency of INH-resistance among Beijing genotype strains was significantly lower than that among non-Beijing genotype strains (*p* = 0.032). In addition, the Beijing genotype family readily formed clusters.

**Conclusions:**

Our findings indicate that male and middle-aged people were more probably be infected by Beijing genotype *M.tb*, older people and non-residents were more probably be infected by non-Beijing genotype *M.tb*. The high percentage of resistance to INH occurring in non-Beijing genotype strains suggested that non-Beijing genotype strains should be given much more interest in Beijing.

**Electronic supplementary material:**

The online version of this article (10.1186/s12879-018-3578-7) contains supplementary material, which is available to authorized users.

## Background

Tuberculosis (TB) is an infectious disease and a significant global public health problem [1, 2]. There were an estimated 10.1 million incident TB cases worldwide in 2017 [3], of which about 6.4 million were notified. Of these, there were an estimated 558,000 cases of multidrug-resistant TB (MDR-TB) and rifampicin resistant TB (RR-TB) [3]. China is one of the 30 high TB burden countries. Although the TB incidence rate and the case fatality ratio have fallen, TB continues to be a public health problem in China [3]. TB is also a public health problem in Beijing, the capital of the People’s Republic of China [4]. There is a need for more studies to clarify the association between drug resistance and genotype diversity in Beijing, in particular to, investigate the relationship between drug resistance and Beijing genotype family strains.

Molecular biology tools have been used extensively to determine the genotypic diversity of *M.tb* strains, and have played an important role in TB control and understanding TB epidemiology over the last few decades [5–7]. Spoligotyping and Variable number of tandem repeat (VNTR) are considered to be powerful tools for differentiating the *M.tb* complex into various genotypes [8, 9].While there are many different *M.tb* genotypes, *M.tb* Beijing genotype strains have attracted global attention because of their wide geographical distribution [10]. Beijing genotype strains were first described by van Soolingen et al. in 1995, and were named after Beijing as they were isolated there [11]. Later, the Beijing genotype was also detected in other regions of the world, especially in East Asia [12–14]. Beijing genotype strains are generally considered to be correlated with community outbreaks of TB [15], escaping from the protective effect of the BCG vaccine [16], efficient dissemination or increased virulence [17], and an increased risk on the occurrence of drug resistance [18, 19].

It is very important to have a clearer understanding of the association between the prevalence of Beijing genotype *M.tb* and resistance to anti-TB drugs. Studies to date have not come to consistent conclusions. While some studies have suggested that development of drug resistance in *M.tb* is associated with its genotype [8, 20], drug resistance more likely occurring among Beijing genotype strains [19, 21, 22], and that Beijing genotype strains are more probably develop MDR-TB [23] and be clustered [24], different results have been obtained in different cities and regions [7, 25]. It would appear that the conclusion drawn from a particular study depends upon the project setting considered, the types of drugs considered and the geographical distribution of strains [26].

Beijing is confronting public health issues in the prevention and control of TB [4, 27]. The association between the prevalence of Beijing genotype strains and their drug resistance status in Beijing is unclear. Studies to date have been hindered by many problems, including the fact that the *M.tb* strains chosen were not sufficiently representative, or the number of strains included in was relatively low [21, 22, 28]. It is thus necessary to clarify the situation, particularly considering the high prevalence of tuberculosis and the inconsistent conclusions obtained from different regions and laboratories.

To clarify if there is an association between the Beijing genotype and resistance to anti-TB drugs, we investigated drug susceptibility patterns in Beijing genotype and non-Beijing genotype strains of *M.tb* strains in Beijing, comparing differences in patterns of drug resistance, and analyzing the relationship between genotype patterns and DST results. This study provides important insight into the association between Beijing genotype family strains and resistance to first-line anti-TB drugs in Beijing.

## Methods

### Description of isolated strains

The 1189 *M.tb* strains analyzed in this study were obtained from pulmonary TB patients, whose sputum was culture-positive for *M.tb.* Strains were collected in 2009 from all districts of Beijing and delivered to the Beijing Tuberculosis and Thoracic Tumor Research Institute [4]. Protocols used in this study were approved by the Ethics Committee of the Beijing Tuberculosis and Thoracic Tumor Research Institute. Patients were included once they signed an informed consent form.

### Spoligotyping

Spoligotyping was performed using the standard protocol [29], and was used to identify the genotype of TB strains according to direct repeat (DR) locus as described previously [4]. Both typical Beijing and Beijing-like genotypes were considered to belong to the Beijing genotype. A commercially available kit (SPOLIGOTYPING KIT, Isogen Bioscience BV, Maarssen, The Netherlands) was used according to the manufacturer’s instructions. The membrane was detected with a chemiluminescence system, using ECL detection liquid (Amersham, Buckinghamshire, United Kingdom) and X-OMAT film (Kodak, Rochester, NY, US) [4].

### VNTR typing

Primers used for amplification of the seven MIRU loci (MIRU-10, 16, 20,23,31,39 and 40), two QUB loci (QUB-11b and QUB-4156C) and three Mtub loci (Mtub04, Mtub21, Mtub24) are listed in Additional file 1: Table S2. VNTR typing was carried out as described previously [4].

### Drug susceptibility testing

Drug susceptibility testing (DST) of the *M.tb* strains to four first-line anti-TB drugs (Isonazid, Rifampin, Ethambutol, and Streptomycin) was performed using the proportion method, as recommended by WHO/IUATLD [30]. Concentrations of drugs in L-J media were as follows: isoniazid (INH) 0.2 μg/mL, rifampin (RFP) 40 μg/mL, ethambutol (EMB) 2 μg/mL and streptomycin (SM) 4 μg/mL [22]. Strains were considered resistant to drugs when the growth rate exceeded 1% compared to the control strain [9]. H37Rv strain was used as a control strain and was tested each batch of DST. Strains which showed resistance to isoniazid and rifampin were defined as MDR-TB. All drugs were purchased from Sigma-Aldrich (St. Louis, MO).

### Data management

Spoligotyping data and VNTR data were analyzed using BioNumerics software (Version 5.0, Applied-Maths, Sint-Martens-Latem, Belgium) [9]. All spoligotyping data were compared with the SITVIT_WEB database (http://www.pasteur-guadeloupe.fr:8081/SITVIT_ONLINE/) [31]. The Hunter-Gaston discriminatory index (HGDI) of each genotyping method was calculated according to the previously published article [32]. The percentage clustering was calculated as (*n*_*c*_-*C*)/*N*, where *N* is the total number of isolates, *C* is the number of clusters, and *n*_*c*_ is the total number of clustered isolates [27, 33].

### Statistical analysis

Statistical calculations were performed in SPSS 21 software. Chi-square or Fisher’s exact probability tests were used to compare the proportions of different groups [9]. A *p*-value less than 0.05 was considered statistically significant. Odd ratios (ORs) and 95% confidence intervals (CI) were calculated to measure the association between genotype and DST results [27].

## Results

### The characteristics of studied population

**Fig. 1:**
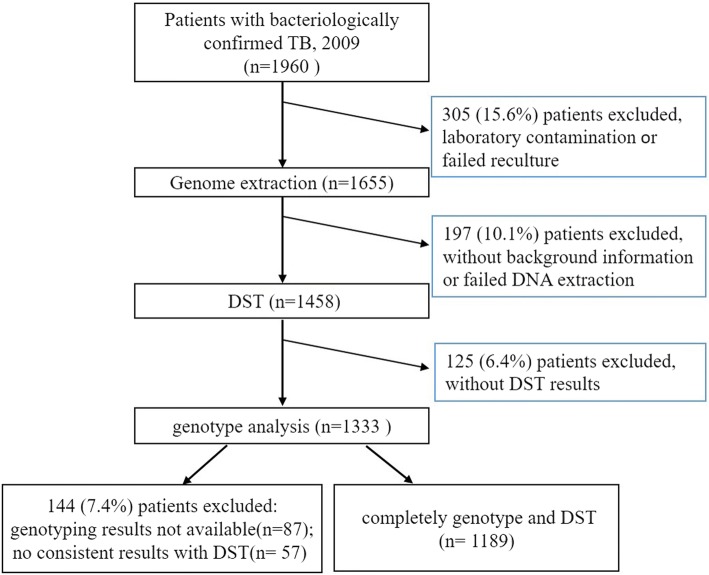
The study population examined in this study and the study workflow

Basic information of the 1189 individual *M.tb* strains collected from Beijing were analyzed (Fig. 1). All isolates investigated in this study were classified according to patient’s age and gender (Table 1). The study population included 819 men (76.9%) and 370 women (23.1%), with a median age of 35 (range from 13 to 92) years, of whom 651 were Beijing residents and 538 were non-residents (Table 1). These data suggest that the resident population and non-resident population made an equal contribution to TB prevalence in the population studied.

**Table 1:** The background information of 1189 patients

Characteristic	Variable	Number of reported cases	Beijing family (%)	Non-Beijing Family (%)	OR (95% CI)	*p* value
All		1189	990	199		
Sex	Male	819	695(70.2)	124(62.3)	1.127(1.004–1.264)	0.018
	Female	370	295(29.8)	75(37.7)		
Age groups, years	< 25	296	242(24.5)	54(27.1)	0.901(0.7–1.16)	0.237
	25–44	462	389(39.3)	73(36.7)	1.071(0.878–1.306)	0.272
	45–64	261	229(23.1)	32(16.1)	1.438(1.027–2.015)	0.016
	≥65	170	130(23.1)	40(20.1)	0.653(0.474–0.9)	0.005
History of TB	New cases	1065	885(89.4)	180(90.5)	0.981(0.909–1.063)	0.383
	Treatment	124	105(10.6)	19(9.5)	1.111(0.698–1.767)	
Registered residence	Residence	651	530(53.5)	121(60.8)	0.880(0.776–0.998)	0.035
	Non-residence	538	460(46.5)	78(39.2)	1.185(0.985–1.427)	

*p* value indicates whether there is a significant difference between Beijing family and non-Beijing family in sex, age, household, and history of TB. (*p <* 0.05 represents a statistically significant difference)

### The genotyping structure of *M.tb* strains

To investigate the genotype of the prevalent strains, the 1189 individual *M.tb* strains were analyzed by spoligotyping and VNTR methods. Of these strains, Beijing genotype families accounted for 83.3% (990/1189) (94.7% were belonged to typical Beijing families and 5.3% were belonged to atypical Beijing families), non-Beijing genotype families accounted for 16.7% (199/1189) (Additional file 2: Table S1). The non-Beijing genotype family mainly included the T1 (SIT53), T2 (SIT52) and MANU2 (SIT54) families et al. Furthermore, a newly discovered genotype cluster included forty-two (3.5%) *M.tb* strains.

Allelic diversity among the 1189 strains was calculated for each VNTR locus using the HGDI (Additional file 1: Table S2). Beijing genotype strains showed a significantly higher clustering rate than the non-Beijing genotype strains after cluster analysis, no matter using spoligotyping or VNTR genotyping data (Table 2).

**Table 2:** Comparison of clustered and individual strains between the Beijing and non-Beijing family

Genotyping	Genotype	Beijing family(*n* = 990)	Non-Beijing family(*n* = 199)	*p* value
Spoligotyping	Individual strains, n	10	58	
	Clustered strains, n	980	141	< 0.001
	Clusters, n	11	22	
	Clustering rate, %	98.9	70.8	
VNTR	Individual strains, n	765	181	
	Clustered strains, n	225	18	< 0.001
	Clusters, n	68	8	
	Clustering rate, %	22.7	9	

(*p <* 0.05 represents a statistically significant difference)

### Association with infection by Beijing genotype strains

There were almost three times as many men as women in the study population (ratio = 2.21; 819/370), and Beijing genotype family strains showed higher prevalence in men (ratio = 2.36; 695/295; *p* = 0.018) (Table 1). We found that Beijing genotype *M.tb* strains were more probably be isolated from men [*p* = 0.018, OR (95%CI):1.127(1.004–1.264)]and people in the 45–64 age group [*p* = 0.016, OR (95% CI): 1.438 (1.027–2.015)]. These results suggest that being male or middle aged is associated with infection by Beijing genotype strains. Conversely, non-Beijing genotype *M.tb* strains were more probably be isolated from people who are older than 65 [*p* = 0.005, OR (95%CI): 0.653 (0.474–0.9)] or non-resident [*p* = 0.035, OR (95%CI): 1.185(0.985–1.427)], indicating that being older or non-resident is associated with infection by non-Beijing genotype strains.

Different association factors were obtained when data from the pan-susceptible TB and drug resistant TB groups were analyzed separately (Table 3). In all the pan-susceptible TB groups, males [*p* = 0.035, OR (95%CI): 1.127(0.980–1.295)] and those aged 45–64 [*p* = 0.021, OR (95%CI): 1.556(1.001–2.419)] were more probably be infected with Beijing genotype strains, consistent with the results above. In all the drug resistant TB groups, results showed that the over 65 s [*p* < 0.001, OR (95%CI): 0.254(0.139–0.465)] and non-residents [*p* = 0.002, OR (95%CI): 0.703(0.577–0.856)] were more probably be infected with non-Beijing genotype strains, indicating that being older and non-resident are association factors for infection with the non-Beijing genotype.

**Table 3:** The General demographic characteristics difference of Beijing genotype family and non-Beijing family patients among the Pan-susceptible TB and any drug resistant TB

Characteristic	Category	Pan-susceptible TB					Any drug resistant TB				
		Number of reported cases	Beijing family (%)	Non-Beijing family (%)	OR (95% CI)	*p* value	Number of reported cases	Beijing family (%)	Non-Beijing family (%)	OR (95% CI)	*p* value
All		849	710	139			340	280	60		
sex	Male	581	495(69.7)	86(61.9)	1.127(0.980–1.295)	0.035	238	200(71.4)	38(63.3)	1.128(0.918–1.386)	0.107
	Female	268	215(30.3)	53(38.1)			102	80(28.6)	22(36.7)		
Age, years	< 25	226	182(25.6)	44(31.7)	0.810(0.615–1.066) 1.001(0.792–1.267)	0.071	70	60(21.4)	10(16.7)	1.286(0.7–2.363) 1.255(0.868–1.816)	0.242
		318	266(37.5)	52(37.4)		0.445	144	123(43.9)	21(35)		0.125
	25–44										
	45–64	170	151(21.3)	19(13.7)	1.556(1.001–2.419)	0.021	91	78(27.9)	13(21.7)	1.286(0.767–2.156)	0.211
	≥65	135	111(15.6)	24(17.3)	0.905(0.606–1.354)	0.315	35	19(6.8)	16(26.7)	0.254(0.139–0.465)	< 0.001
History TB	New cases	800	667(94)	133(95.7)	0.982(0.943–1.022) 1.403(0.609–3.232)	0.216	265	218(77.9)	47(78.3)	0.994(0.858–1.151) 1.022(0.602–1.734)	0.544
	Retreated case	49	43(6)	6(4.3)			75	62(22.2)	13(21.7)		
registered residence	Residence	467	389(54.8)	78(56.1)	0.976(0.831–1.147) 1.030(0.840–1.264)	0.424	184	141(50.4)	43(71.7)	0.703(0.577–0.856) 1.752(1.152–2.665)	0.002
	Non-residence	382	321(45.2)	61(43.9)			156	139(49.7)	17(28.3)		

*p* value indicates whether there is a significant difference between Beijing family and non-Beijing family

(*p <* 0.05 represents a statistically significant difference)

### Difference in drug resistance patterns

DST experiments were carried out on the 1189 strains to investigate the drug-resistance profile of prevalent strains. Results showed that 849 isolates (71.4%) were sensitive to the four first-line anti-TB drugs and 340 isolates (38.6%) were resistant to at least one of the drugs. The percentage of overall drug resistance (resistance to any anti-TB drug) was 38.6%, and that of MDR-TB was 9%. 82.4% (280/340) of the drug resistant *M.tb* strains belonged to the Beijing genotype family. The percentage of strains resistant to INH/ RFP/SM/EMB among Beijing genotype family strains (16.3%/10%/18.7%/13%, respectively) was lower than that among non-Beijing strains (22.1%/10.1%/20.6%/16.1%, respectively).

In this study, the percentage of drug resistance among new TB cases was significantly lower than that in patients undergoing retreatment (Additional file 3: Table S3), suggesting the possibility that improper use of antibiotics, and/or poor treatment compliance among TB patients, may have led to an increase in the occurrence of drug resistance. We compared the percentage of drug resistant strains between Beijing and non-Beijing genotype family strains; the only difference observed was that the percentage of INH-resistance in Beijing genotype strains was significantly lower than that in non-Beijing genotype family strains [16.3% vs 22.1%, OR (95% CI): 0.736(0.547–0.989), *p* = 0.032] (Table 4). We also looked for differences between new (*n* = 1065) and retreated TB cases (*n* = 124) group (Table 4); a similar result was observed [12.4% vs 17.2%, OR (95% CI): 0.722(0.501–1.040), *p* = 0.042] in the new cases group (Table 4). However, statistical analysis did not reveal any difference in these characteristics between Beijing family and non-Beijing family in retreatment TB cases. Our study has revealed a relatively high prevalence of MDR-TB in Beijing, but does not indicate any significant association between MDR-TB and the Beijing genotype family.

**Table 4:** The difference of drug susceptibility between Beijing genotype family and non-Beijing genotype family when considered first-line drug resistance (*n* = 1189)

Characteristic	Category	Total					New cases					Retreated cases				
		Number of reported cases	Beijing family (%)	Non-Beijing family (%)	OR (95% CI)	*p* value	Number of reported cases	Beijing family (%)	Non-Beijing family (%)	OR (95% CI)	*p* value	Number of reported cases	Beijing family (%)	Non-Beijing family (%)	OR (95% CI)	*p* value
All		1189	990	199			1065	885	180			124	105	19		
DST profile	Pansusceptible	849	710(71.7)	139(69.9)	1.027(0.930–1.134)	0.325	800	667(75.4)	133(73.9)	1.020(0.928–1.121)	0.348	49	43(41)	6(31.6)	1.297(0.644–2.613)	0.221
	INH	205	161(16.3)	44(22.1)	0.736(0.547–0.989)	0.032	141	110(12.4)	31(17.2)	0.722(0.501–1.040)	0.042	64	51(48.6)	7(36.9)	1.318(0.709–2.453)	0.173
	RIF	119	99(10)	20(10.1)	0.995(0.631–1.569)	0.534	64	53(6)	11(6.1)	0.980(0.522–1.839)	0.475	55	46(43.8)	9(47.4)	0.925(0.549–1.557)	0.337
	SM	226	185(18.7)	41(20.6)	0.907(0.670–1.227)	0.295	176	143(16.2)	33(18.3)	0.881(0.625–1.242)	0.237	50	42(40)	8(42.1)	0.950(0.534–1.692)	0.431
	EMB	161	129(13)	32(16.1)	0.810(0.568–1.157)	0.151	117	92(10.4)	25(13.9)	0.748(0.496–1.130)	0.086	44	37(35.2)	7(36.9)	0.956(0.503–1.820)	0.447
	MDR	107	88(8.9)	19(9.6)	0.931(0.581–1.492)	0.427	58	48(5.4)	10(5.6)	0.976(0.503–1.893)	0.473	49	40(38.1)	9(47.4)	0.804(0.472–1.370)	0.224

*INH* Isoniazid, *LFP* Rifampicin; *SM* Streptomycin; *EMB* Ethambutol; *MDR* multi-drug resistance

*p* value indicates whether there is a significant difference between Beijing family and non-Beijing family (*p <* 0.05 represents a statistically significant difference)

## Discussion

As far as we know, this is a large-scale study to assess the prevalence of the Beijing genotype *M.tb* strains in Beijing and their association with drug resistance. The Beijing genotype family of *M.tb* is one of the most successful lineages in the present global tuberculosis epidemic. In order to understand how drug-resistant TB develops and to find better ways to control TB, it is essential to understand the molecular epidemiology of *M.tb*. Prior to this study, however, the prevalence of Beijing strains and their drug resistance status in Beijing, the city after which they were named, was unclear.

To examine these problems, we made use of strain collection containing 1189 isolates from a previous project in Beijing [4]. We described the detailed population structure of both Beijing and non-Beijing genotype strains, and investigated the association between the prevalence of Beijing genotype strains of *M.tb* and drug resistance in Beijing.

It has often been found that the prevalence of TB is higher in men than in women [32]. Consistent with results of previous studies [21, 34], we found that men are more probably be infected by *M.tb;* in the pan-susceptible group, men were more probably be infected by Beijing genotype *M.tb* strains (Table 3). As this the result may be affected by the gender ratio in the local population [31], we examined the overall gender ratio in Beijing. As the gender ratio in Beijing was 1.07 to1 (male: female) in 2010 [35], it is likely that the reason for the difference observed is due to differences in susceptibility to *M.tb* among local populations.

With the exception of gender, age was an association factor for infection by the Beijing genotype. A previous study demonstrated that younger patients (aged under 25 years) were likely to be infected with the Beijing genotype [21]. However, we did not observe such a tendency. Instead, we observed that individuals in the middle-aged group were more probably be infected with Beijing genotype strains of *M.tb,* and elderly people were more probably be infected with non-Beijing genotype strains. These results may reflect the possibility that middle-aged and older population groups in Beijing may have different social networks and work situations, suggesting that the population of different region could confront different risks. Further research is required to clarify this hypothesis. Except for age, we found that the non-resident population was more probably be infected by non-Beijing genotype strains of *M.tb*. Non-residents tend to live in areas of Beijing where the population density is high and work in crowded public environments. Non-Beijing genotype *M.tb* strains probably likely spread more readily in these districts.

Research on molecular epidemiology has greatly improved our understanding of TB prevalence. A study from Shanghai has also suggested that Beijing genotype strains are associated with recent transmission of TB and are more probably be clustered [36] [24]. Consistent with this finding, we found that Beijing genotype strains in the study population were significantly associated with clustering, suggesting recent transmission mechanisms are likely significantly different from those of non-Beijing genotype strains. Moreover, VNTR method separated the *M.tb* Beijing genotype strains into much more branches and much smaller clusters, suggesting that it possessed a higher discriminatory power than the spoligotyping method [27].

Evidence has shown that drug resistance in *M.tb* is more probably occur among Beijing genotype strains [19, 21, 22]. However, the nature of the association found between drug resistance and the Beijing genotype has varied between studies, [7, 25]. When we analyzed the percentage of drug resistant strains in different genotypes, results indicated that there was an association between the occurrence of INH resistance and the prevalence of non-Beijing genotype strains. In regard to MDR-TB, some studies have suggested that Beijing genotype strains are more probably develop MDR-TB [23]. Here, however, our data did not support this hypothesis. Our data also revealed a relatively high prevalence of MDR-TB in Beijing, but did not show a significant difference in the percentage of MDR-TB between Beijing and non-Beijing genotypes, suggesting that there is no significant association between the occurrence of MDR-TB and the prevalence of Beijing genotype strains.

According to previous reports and this study, the association between the occurrence of drug-resistance and the prevalence of Beijing genotype strains is variable [19–21]. Possible explanations for the variability include: 1) the number and percentage of Beijing genotype strains included in a study influences the results [36, 37]; 2) variation in the frequency of strains with different drug resistance patterns is influenced by differences in treatment regimens and tuberculosis control programs in different regions [38], as well as characteristics of the host population, socioeconomic factors, or any combination of these factors [39]; and 3) the uneven geographical distribution of the Beijing genotype will also influence the association [40, 41]. Further detailed studies on the association are needed.

While our results are important, this study has some limitations. The association between genotypic diversity and drug resistance is a very complex problem, and research that bears additional factors and underlying reasons in mind is required [37]. Although no selection bias was detected in the demographic characteristics of our study population, there is a higher incidence of serious TB cases in this region, which may have led to an overestimation of drug-resistant TB. In addition, we used traditional Spoligotyping and VNTR methods; better methods may be available used in the future to identify and analyze the Beijing genotype. With developments in science and technology, whole genome sequencing (WGS) can provide greater precision than traditional genotyping for studying the recent transmission of *M.tb* [38]. Nevertheless, spoligotyping and VNTR are still valuable tools for identifying genotypes and performing cluster analysis [39–42]. Each technique has its own advantages and disadvantages and we will combine classical genotyping, WGS, and epidemiological investigations in future studies.

## Conclusions

In summary, our results suggest that male and middle-aged patients in the study population were more probably infected by Beijing genotype *M.tb* strains, and elderly patients and non-residents were more likely to be infected by non-Beijing genotype *M.tb*. The Beijing genotype was prevalent in this study population, and Beijing genotype strains readily formed clusters. Our data also indicates that there is a higher percentage of INH-resistant in the middle of non-Beijing genotype family than that among Beijing genotype family *M.tb* strains in Beijing. This study provides important insights into the prevalence of *M.tb* strains in Beijing.

## Additional files

Additional file 1: **Table S2.** The Hunter-Gaston discriminatory index of the 12 VNTR loci in M.tb strains from Beijing. (DOCX 16 kb)

Additional file 2: **Table S1.** Spoligotyping patterns result of M.tb strains collected from Beijing in this study. (DOCX 15 kb)

Additional file 3: **Table S3.** Drug susceptibility phenotypes of new cases and retreatments. (DOCX 14 kb)

Additional file 4: Detailed information about genotyping patterns and DST results isolated from 1189 M.tb strains in Beijing. (XLSX 123 kb)

## Abbreviations

CI: Confidence intervals
DST: Drug susceptibility testing
EMB: Ethambutol
HGDI: Hunter-Gaston discriminatory index
INH: Isoniazid
*M.tb*: *Mycobacterium tuberculosis*
MDR: Multidrug-resistant
ORs: Odd ratios
RFP: Rifampin
SM: Streptomycin
TB: Tuberculosis
VNTR: Variable number of tandem repeat
WHO: World Health Organization
XDR: Extremely drug-resistant
