# Supplementary material for: Characterization of Mycobacterium tuberculosis strains in Beijing, China: drug susceptibility phenotypes and Beijing genotype family transmission
Source: BMC Infect Dis. 2018 Dec 14;18:658. doi: 10.1186/s12879-018-3578-7 (PMC6295058; doi:10.1186/s12879-018-3578-7)
Supplement: Supplementary file 1 — Table S2. The Hunter-Gaston discriminatory index of the 12 VNTR loci in M.tb strains from Beijing. (DOCX 16 kb) [file 12879_2018_3578_MOESM1_ESM.docx]

Table S2. The Hunter-Gaston discriminatory index of the 12 VNTR loci in M. tuberculosis strains from Beijing.

| Order | VNTR locus | VNTR alias | No. of alleles | Allelic diversity (h*) for | | | | | | | |
| --- | --- | --- | --- | --- | --- | --- | --- | --- | --- | --- | --- |
|  |  |  |  | Beijing family(n=990) | |  | Non-Beijing family(n=199) | |  | All strains (n=1189) | |
|  |  |  |  | Individual locus | cumulative |  | Individual locus | cumulative |  | Individual locus | cumulative |
| 1 | 0424 | Mtub04 | 7 | 0.6236 | 0.6236 |  | 0.6579 | 0.6579 |  | 0.6319 | 0.6319 |
| 2 | 1955 | Mtub21 | 5 | 0.5962 | 0.7198 |  | 0.6865 | 0.7768 |  | 0.5939 | 0.7585 |
| 3 | 2074 | Mtub24 | 5 | 0.4847 | 0.7986 |  | 0.4705 | 0.8163 |  | 0.4958 | 0.8019 |
| 4 | 2163 | QUB-11b | 7 | 0.7155 | 0.8766 |  | 0.7365 | 0.8901 |  | 0.7455 | 0.8792 |
| 5 | 4156 | QUB-4156c | 7 | 0.2234 | 0.8895 |  | 0.2959 | 0.8964 |  | 0.2409 | 0.8924 |
| 6 | 0960 | MIRU10 | 6 | 0.4696 | 0.9158 |  | 0.6832 | 0.9378 |  | 0.6394 | 0.9247 |
| 7 | 1644 | MIRU16 | 7 | 0.6385 | 0.9566 |  | 0.7237 | 0.9693 |  | 0.6549 | 0.9633 |
| 8 | 3007 | MIRU20 | 3 | 0.2654 | 0.9613 |  | 0.2983 | 0.9794 |  | 0.2618 | 0.9712 |
| 9 | 2531 | MIRU23 | 3 | 0.1187 | 0.9775 |  | 0.1591 | 0.9868 |  | 0.1485 | 0.9806 |
| 10 | 3192 | MIRU31 | 8 | 0.5421 | 0.9935 |  | 0.6438 | 0.9988 |  | 0.5145 | 0.9967 |
| 11 | 4348 | MIRU39 | 5 | 0.2784 | 0.9983 |  | 0.4519 | 0.9991 |  | 0.3368 | 0.9987 |
| 12 | 0802 | MIRU40 | 7 | 0.3741 | 0.9984 |  | 0.5984 | 0.9993 |  | 0.4869 | 0.9990 |

* h, Hunter-Gaston discriminatory index.
