# Supplementary material for: Characterization of Mycobacterium tuberculosis strains in Beijing, China: drug susceptibility phenotypes and Beijing genotype family transmission
Source: BMC Infect Dis. 2018 Dec 14;18:658. doi: 10.1186/s12879-018-3578-7 (PMC6295058; doi:10.1186/s12879-018-3578-7)
Supplement: Supplementary file 2 — Table S1. Spoligotyping patterns result of M.tb strains collected from Beijing in this study. (DOCX 15 kb) [file 12879_2018_3578_MOESM2_ESM.docx]

Table S1. Spoligotyping patterns result of *M. tuberculosis* strains collected from Beijing in this study.

^$^ The number of collected strains isolates.

* 990(83.3%) strains belonged to Beijing families.

^#^ 199(16.7%) strains (include 16 newfound strains) belonged to non-Beijing families.

| No.^$^ | % (No.) | | | |  |  | | |  | | | | | | | | |
| --- | --- | --- | --- | --- | --- | --- | --- | --- | --- | --- | --- | --- | --- | --- | --- | --- | --- |
|  | **Beijing families*** | |  |  | | | **non-Beijing families^#^** | | | | | | | | | | |
|  | Typical  Beijing | Atypical  Beijing |  | T1 | | | | T2 | | T3-4 | MANU 2 | MANU 3 | U | H3 | CAS | BCG | New found |
| 1189 | 78.8%  (937) | 4.5%  (53) |  | 4.6%  (55) | | | | 2.1%  (25) | | 8 | 4.5%  (54) | 2 | 8 | 2 | 2 | 1 | 3.5%  (42) |
