# Supplementary material for: Characterization of Mycobacterium tuberculosis strains in Beijing, China: drug susceptibility phenotypes and Beijing genotype family transmission
Source: BMC Infect Dis. 2018 Dec 14;18:658. doi: 10.1186/s12879-018-3578-7 (PMC6295058; doi:10.1186/s12879-018-3578-7)
Supplement: Supplementary file 3 — Table S3. Drug susceptibility phenotypes of new cases and retreatments. (DOCX 14 kb) [file 12879_2018_3578_MOESM3_ESM.docx]

Table S3. Drug susceptibility phenotypes of new cases and retreatments.

| Characteristic | Category | Number of reported cases | New cases (%) | Retreated cases (%) | OR (95% CI) | *p* Value |
| --- | --- | --- | --- | --- | --- | --- |
| All |  | 1189 | 1065 | 124 |  |  |
| DST profile | Pansusceptible | 849 | 800(67.3) | 49(39.5) | 1.901(1.525-2.370) | <0.001 |
|  | INH | 205 | 141(13.2) | 64(51.6) | 0.257(0.204-0.323) | <0.001 |
|  | RIF | 119 | 64(6) | 55(44.4) | 0.135(0.1-0.184) | <0.001 |
|  | SM | 226 | 176(16.5) | 50(40.3) | 0.410(0.318-0.528) | <0.001 |
|  | EMB | 161 | 117(11) | 44(35.5) | 0.310(0.231-0.415) | <0.001 |
|  | MDR | 107 | 58(5.5) | 49(39.5) | 0.138(0.099-0.192) | <0.001 |

INH: Isoniazid; LFP: Rifampicin; SM: Streptomycin; EMB: Ethambutol; MDR: multi-drug resistance.

*p* value indicates whether there is a significant difference between Beijing family and non-Beijing family (*p<*0.05 represents a statistically significant difference).
